# Activatable Peptides for Rapid and Simple Visualization of Protease Activity Secreted in Living Cells

**DOI:** 10.3390/ijms23031605

**Published:** 2022-01-30

**Authors:** Gae-Baik Kim, Jeong Min Lee, Duc Long Nguyen, Joonseok Lee, Young-Pil Kim

**Affiliations:** 1 Department of Life Science, Hanyang University, Seoul 04763, Korea; valkyriex@naver.com (G.-B.K.); jeremy1989@naver.com (J.M.L.); quyle.dongphuong@gmail.com (D.L.N.); 2 Research Institute for Convergence of Basic Sciences, Hanyang University, Seoul 04763, Korea; 3 Department of Chemistry, Hanyang University, Seoul 04763, Korea; joonseoklee@hanyang.ac.kr; 4 Department of HY-KIST Bio-Convergence, Hanyang University, Seoul 04763, Korea; 5 Molecular Recognition Research Center, Korea Institute of Science & Technology (KIST), Seoul 02792, Korea; 6 Research Institute for Natural Sciences, Hanyang University, Seoul 04763, Korea

**Keywords:** activatable peptides, fluorescent imaging, proteases, MMP2, cancer cells

## Abstract

Activity-based monitoring of cell-secreted proteases has gained significant interest due to the implication of these substances in diverse cellular functions. Here, we demonstrated a cell-based method of monitoring protease activity using fluorescent cell-permeable peptides. The activatable peptide consists of anionic (EEEE), cleavable, and cationic sequences (RRRR) that enable intracellular delivery by matrix metalloproteinase-2 (MMP2), which is secreted by living cancer cells. Compared to HT-29 cells (MMP2-negative), HT-1080 cells (MMP2-positive) showed a strong fluorescence response to the short fluorescent peptide via cell-secreted protease activation. Our approach is expected to find applications for the rapid visualization of protease activity in living cells.

## 1. Introduction

Rapid monitoring of protease-initiated substrate-specific proteolysis in cells is essential for understanding many biological functions, as proteases have been dynamically implicated in cancer, inflammation, and degenerative diseases [1,2,3]. Cell-secreted proteases such as matrix metalloproteases (MMPs) mediate matrix degradation, wound healing, and tumor invasion in the extracellular matrix (ECM); monitoring the activities of cell-secreted proteases is thus attractive for tissue remodeling and anti-tumoral strategies [4,5,6]. Over the past decade, a number of approaches for monitoring MMP activity have been reported both in vitro and in vivo [7,8,9,10,11]. In particular, protease-activatable cellular imaging has been developed using fluorescent polymers [12] and nanoparticles [13] in conjunction with peptide substrates. However, despite their widespread development, these probes are limited to general use in biological laboratories due to the complicated processes required to synthesize them and their multivalent nature that is often difficult to control.

Here, we report a simple and rapid method of monitoring cell-secreted MMP activity using fluorophore-labeled cell-permeable peptides. Unlike dye-to-quencher peptide substrates designed to visualize MMP activity [14,15,16], our short and linear activatable peptides are capable of intracellular localization upon cell-secreted MMP activity. The peptides consist of three functional parts: a cell-permeable portion (the positively charged residue; tetra-arginine; R_4_), a protease-cleavable section, and a part that blocks amino acids (the negatively charged residue; tetra-glutamic acid; E_4_). To allow the intracellular visualization of this probe, a commonly used dye, fluorescein isothiocyanate (FITC), can be used to label cell-permeable amino acids. The activatable concept of the cell permeable peptides has been reported in previous strategies [12,13,17], but fluorescent peptides in this study provide a more straightforward means without the complicated design. It is noteworthy that arginine-rich peptides, known as cell-penetrating peptides (CPPs), can translocate through living cell membranes because cancer cells are distinguished by their negatively charged surfaces due to their altered biosynthetic processes, which result in appended negative molecules such as phospholipids, glycolipids, and glycoproteins [18,19,20]. Within the mechanism that drives CPP translocation into cells, this study focused on protease-activated live-cell imaging through the intracellular uptake of cleavable peptides, where no bioactive cargo, such as proteins, polymers, or nanomaterials [21,22], is conjugated with peptides.

## 2. Results and Discussion

### 2.1. Principle of MMP-Activated Cellular Imaging

**Scheme 1:**
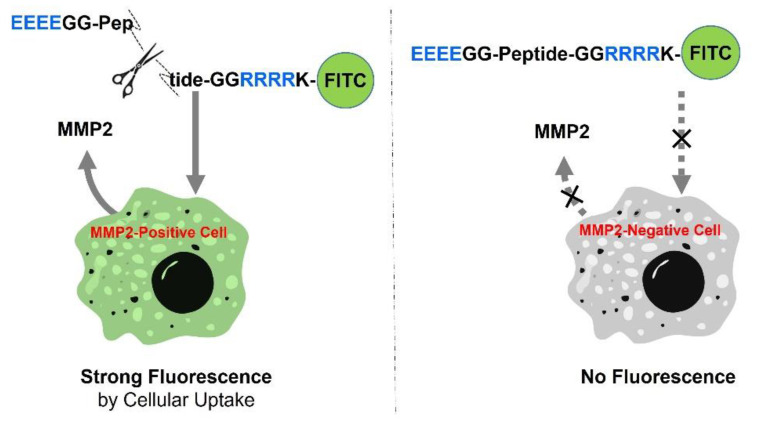
Schematic of peptide-permeable cell imaging in response to MMP2 activity. After treatment with FITC-labeled peptides, an MMP2-positive (MMP2-secreted) cell shows a strong fluorescence due to intracellular delivery of cleaved peptides (**left**), whereas an MMP2-negative cell (**left**) does not generate fluorescence due to the lower cell-permeability of the peptide (**right**).

As depicted in Scheme 1, an activatable peptide substrate (Pep1, EEEEGGIPVSLRSGGGRRRRK-FITC) was used for MMP2-specific intracellular imaging and was comprised of anionic amino acids (EEEE; E_4_), an MMP2-specific substrate sequence (IPVSLRSG; cleavage site between S and L), and cationic amino acids (RRRR; R_4_). Two glycine residues (GG) at the ends of the substrate sequence serve as linkers. In this study, MMP2 and its specific peptide substrate were chosen as a model for cell-secreted protease. MMP2 and MMP9 secretions are elevated in several types of human cancer cells [23,24]. However, compared to the secretion level of MMP9, which is controlled by cytokines or signal transducers, MMP2 is expressed constitutively by most cancer cell lines [23]. Since cell-secreted MMP2 can preferentially degrade this substrate sequence (IPVSLRSG), as reported previously [10,25], a simple linear peptide can monitor the MMP2 secretion activity of cancer cells. In this regime, Pep 1 is activated in the presence of MMP2-positive cells and produces strong fluorescence through intracellular delivery (left image in Scheme 1), whereas it is silenced in the presence of MMP2-negative cells, resulting in no intracellular fluorescence (right image in Scheme 1). The anionic sequences function to block the cationic sequence via electrostatic interaction (unless Pep 1 is cleaved), but the electrostatic bond between E_4_ and R_4_ is broken when Pep1 is cleaved by MMP2, leading to the rapid cellular uptake of the exposed R_4_ sequence. Due to the high salt concentration in cell growth media, it is expected that the cleavage-induced separation between E_4_ and R_4_ cannot be reassociated because the salt bridge created through electrostatic interaction impedes the close proximity of two peptide fragments with opposite charges [26]; this reason facilitates cellular uptake upon the cleavage of the peptide due to the cell permeability of positively charged peptides. Based on the important role of the arginine (R) residue in various CPP sequences [27], we chose the shortest R-rich peptides (R_4_) in model cancer cells as previously reported [13], even though their length, structural diversity with other sequences, and membrane translocation effects have been studied [28]. Consequently, the linkage between R_4_ and FITC allows for the imaging of the MMP2-postive cells, but not the MMP2-negative cells.

### 2.2. Determination of MMP2 Expression in Cells

**Figure 1:**
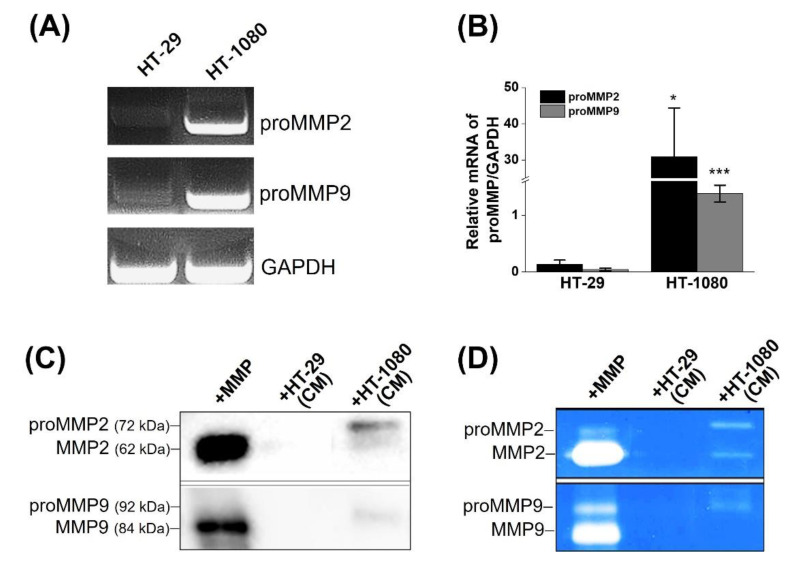
Expression and activity assays of MMP2 and MMP9 in two cell lines (HT-29 and HT-1080). (**A**) Gel electrophoresis and (**B**) real-time PCR showing mRNA expressions of proMMP2 and proMMP9 relative to that of GAPDH in HT-29 and HT-1080. The asterisk denotes significant difference between HT-29 and HT-1080 (* *p* < 0.05, *** *p* < 0.001, Student’s *t* test, *n* = 3). (**C**) Western blot and (**D**) gelatin zymographic images of (pro)MMP2 and (pro)MMP9 in the CM of HT-29 or HT-1080. Active MMP2 or MMP9 enzyme was loaded into the first lane.

Human fibrosarcoma (HT-1080) and human colon cancer (HT-29) cells were used as MMP-positive and MMP-negative cells, respectively. HT-1080 is a malignant cell type that is commonly employed to model cell motility and metastatic progression and demonstrates high MMP expression [29,30]. To evaluate the gene and protein expression levels of MMP2 and MMP9 in both cell lines, we performed RT-PCR, real-time PCR, and Western blotting (Figure 1A–C). In addition, gelatin zymography was performed with the culture media (CM) of the cells to examine the secretion and activity of MMP2 and MMP9 (Figure 1D).

We first investigated the mRNA expression of proMMP2 and proMMP9 in the two cell lines because MMPs are expressed initially as inactive proenzymes, followed by a proteolytic process that leads to their extracellular release as active enzymes. When cDNAs that have been reverse-transcribed from polyadenylated RNA were used for PCR or real-time PCR, the PCR (Figure 1A) and real-time PCR products (Figure 1B) showed significantly higher expression of proMMP2 and proMMP9 in HT-1080 than in HT-29 cells, which were compared to the housekeeping gene (GAPDH) in HT-1080 and HT-29. The proMMP2 expression level in HT-1080 cells was higher than that of proMMP9, indicating the relatively high quantitation of proMMP2 gene expression in HT-1080 cells. The expressed proteins were observed at approximately 66 kDa (active MMP2), 72 kDa (proMMP2), and 92 kDa (proMMP9) in the CM of HT-1080 (Figure 1C), supported by gelatin zymographic results (Figure 1D). In contrast, there was no expression or activity of pro- or active MMP2/9 in the CM of HT-29. This result indicates that MMP2 and MMP9 in HT-1080 were secreted into the CM as latent zymogenic forms, and MMP2 was subsequently transformed into its activated form, which was consistent with the expression and activation patterns reported in many human tumors, including HT-1080 [23,24,31].

### 2.3. Peptide Substrate Specificity for MMP2

**Figure 2:**
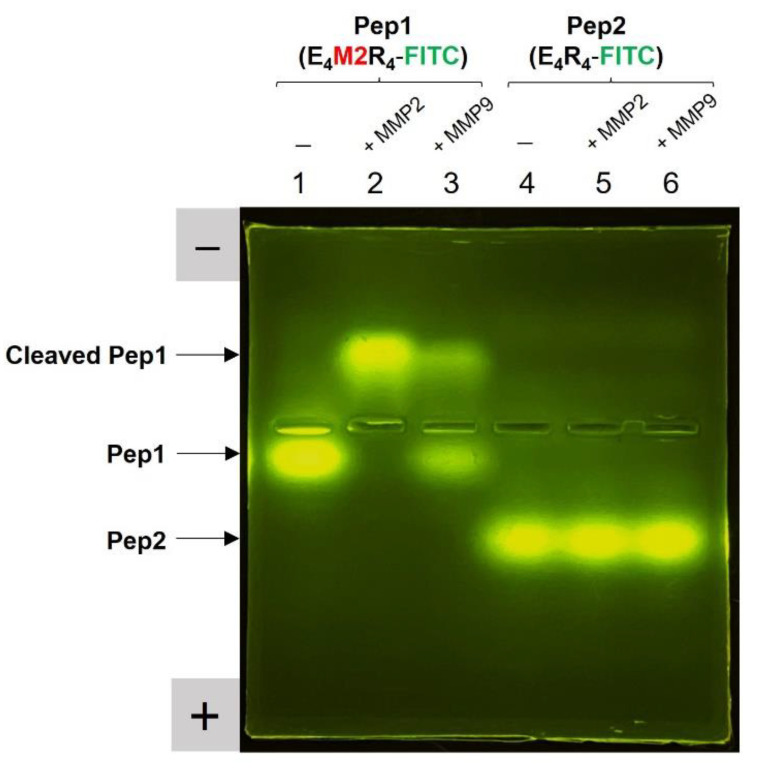
Fluorescent gel image used to verify the cleavage of Pep1 and Pep2 due to MMP activity. Pep1 (lanes 1–3) or Pep2 (lanes 4–6) was loaded onto the agarose gel in the absence of enzymes (lanes 1 and 4) and in the presence of active MMP2 (lanes 2 and 5) or active MMP9 (lanes 3 and 6). The FITC-specific fluorescent band regions are indicated by arrows on the left side of the image, which corresponds to cleaved Pep1, Pep1, and Pep2 (from top to bottom). The agarose gel was exposed to LED irradiation to obtain the FITC-emitted image.

To examine whether Pep1 is cleaved by MMP2 or MMP9, we performed fluorescent gel electrophoresis with Pep1 in the presence or absence of enzymes. Similar to nucleic acids, this peptide-based gel electrophoresis can visualize a distinct difference in charge or length of gel-loaded peptide products under electrophoretic conditions. As shown in Figure 2, when a Pep1 (E_4_M2R_4_-FITC) sample including a substrate sequence (M2; IPVS/LRSG) was treated with active MMP2, a strong fluorescent band shift from Pep1 (E_4_M2R_4_-FITC) to the cleaved Pep1 (LRSGGG-R_4_-FITC) was observed on the agarose gel (lane 2) as compared to the intact fluorescent band of Pep1 (lane 1). The strong fluorescence band upshift was attributed to the change in charge of the peptide by the loss of E4. This result indicates the cleavage of Pep1 into two species (i.e., E_4_-GGIPVS and LRSGGG-R_4_-FITC). The cleavage of Pep1 by MMP9 (lane 3) was less effective than that by MMP2, which suggests that Pep1 has a greater affinity for MMP2 than MMP9. It has been reported that the peptide substrate (IPVSLRSG) has an approximately 7-fold higher *k*_cat_/*K*_M_ for MMP2 (8.2 × 10^4^) than for MMP9 (1.2 × 10^4^) [25]. In contrast, when Pep2 (E_4_R_4_-FITC) without a peptide substrate sequence was used as a negative control, it did not show any fluorescent band shifts on the agarose gel under the same conditions (lanes 4–6), which further supports the presence of enzyme-specific peptide cleavage.

### 2.4. Live Cell Imaging in Response to MMP2 Activity

**Figure 3:**
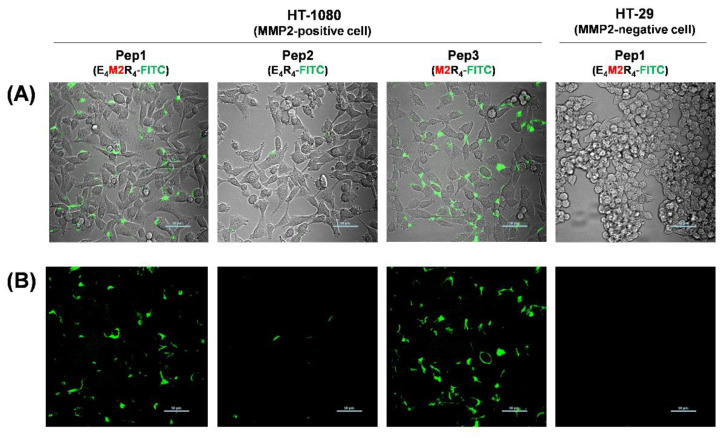
(**A**) Overlay (bright-field and fluorescence) and (**B**) fluorescence confocal images of HT-1080 (MMP2-positive cells) and HT-29 (MMP2-negative cells) treated with different peptides (Pep1, Pep2, or Pep3). A 10 μM peptide was added to the serum-free culture media of the cells. Images were collected from 2 h after treatment. Scale bar: 50 μm.

We also investigated whether Pep1 directly monitors the MMP-2 activity of living cells. HT-1080 (MMP2-positive) and HT-29 (MMP2-negative) cells were cultured in Lab-Tek culture chambers, and different peptides (Pep1, Pep2, or Pep3 in each final 10 μM) were added to serum-free CM during cell growth. When confocal microscopy images were obtained 2 h after peptide treatment in FITC emission channels, strong fluorescence images were observed for Pep1 as well as Pep3 (M2R_4_-FITC, positive control without E4) in HT-1080 (Figure 3A,B). The fluorescence intensity of Pep 1 inside HT-1080 cells was similar to that of Pep3, which indicated that the fluorescence of Pep1 had been caused by substrate cleavage of cell-secreted MMP2. No significant fluorescence images were observed for Pep2 in HT-1080 or for Pep1 in HT-29 cells. When we conducted continuous live cell imaging treated with 10 μM Pep1 for 24 h in serum-free media conditions, the fluorescence intensity decreased after 12 h and was completely lost after 24 h, probably due to the fast degradation of the FITC or its signal attenuation in acidic endosomes (Figure S1). In addition, no cytotoxicity was observed at the particular peptide concentration (final 10 μM) used for 24 h (Figure S2). More importantly, the addition of the MMP2 inhibitor to Pep1 significantly reduced the intracellular fluorescence intensity in MMP2-secreted HT-1080 cells (Figure S3). This result strongly suggests that Pep1 induced rapid intracellular uptake in response to the activity of MMP2 secreted by HT-1080 cells. This R_4_-based cellular uptake was consistent with a previous observation [13]. 

**Figure 4:**
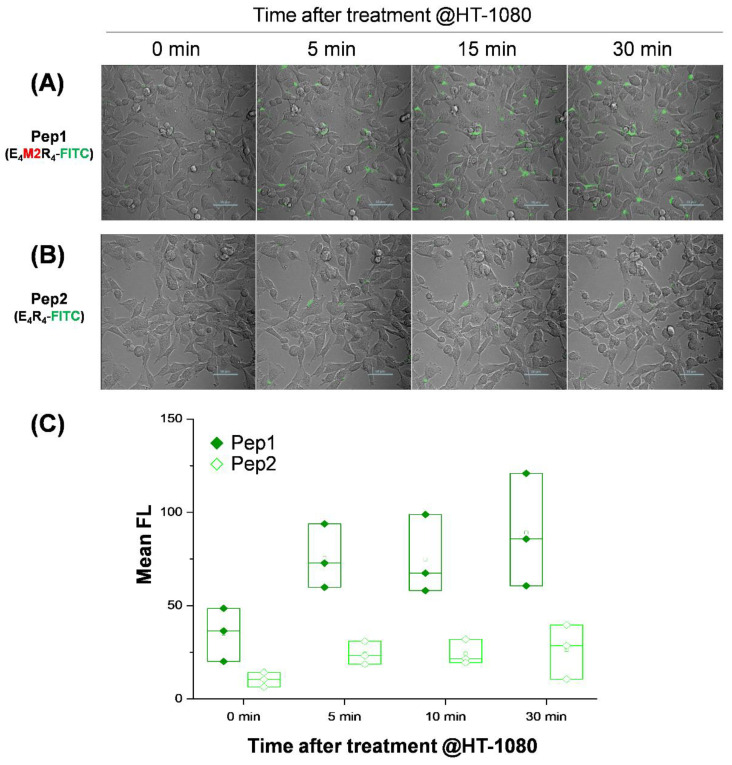
Time-lapse overlay of bright-field and fluorescence images of HT-1080 cells at 37 °C over time (from 0 to 30 min) after peptide treatment. (**A**) Pep1 or (**B**) Pep 2 (final 10 μM each) was added to the CM of HT-1080 cells. Scale bar: 50 μm. (**C**) Box plots showing changes in mean fluorescence (FL) of cells over time in (**A**,**B**) images. The small square dot in the box represents the mean value in triplicate experiments.

**Figure 5:**
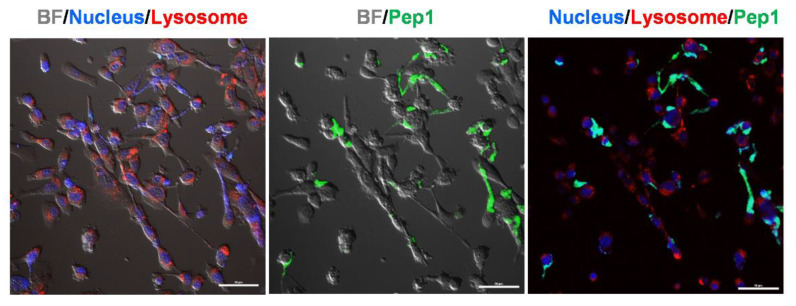
Overlay confocal images of living HT-1080 cells 30 min after Pep1 treatment. Fluorescence images were obtained from the nucleus (blue) by NucBlue, lysosome (red) by Lysotracker Red, and localization of Pep1 (green). BF represents bright-field image. Scale bar: 50 μm.

To further demonstrate the time-lapse response of Pep1 to HT-1080 cells, we obtained fluorescence cell images over time (from 0 to 30 min) after Pep1 treatment (Figure 4). The signal intensity for Pep1 increased even after just 5 min in living HT-1080 cells and became almost saturated (Figure 4A, Movies S1 and S2) as compared to that of the control peptide without a substrate sequence (Pep2) (Figure 4B,C). Figure 4A,B were nearly identical to the images obtained at 2 h in Figure 3A,B, respectively. As observed in Figure 3, the uptake rate of Pep1 was similar to that of Pep3. There was no significant increase in the time-lapse fluorescence of Pep1 in living HT-29 cells for 30 min (Movies S3 and S4). This result indicates that the designed peptide could be very useful for monitoring MMP2 activity in living cells for a relatively short time. In the case of HT-1080 cells treated with Pep1 for 30 min, the fluorescence image treated with a final 10 μM Pep1 was more effective in tracking intracellular uptake than those treated with a final 0.2 μM or 1 μM Pep1 (Figure S4) since this probe caused fast diffusion in the extracellular region across large areas and had a relatively stronger or blurrier intensity than those in the intracellular region under the fluorescence-saturated imaging condition. Based on the signal-to-background intensity and intracellular uptake, the difference in fluorescence intensity between intracellular and extracellular regions (especially in MMP2-expressed cells) was more clearly distinguished at the 10 μM concentration than at the relatively low concentrations (Figure S5). Interestingly, localization of Pep1 in HT-1080 was observed around the cell membrane or in the cytosol rather than in the nucleus or lysosomes (or late endosomes), where Pep1 intensity (green) did not co-localize with nuclei (blue) or lysosomes (red) as a result of staining organelles (Figure 5). Since acidic endosomes can lead to the reduction in FITC fluorescence with a pKa of 6.5, we cannot exclude the possibility that Pep1 could not be observed in late endosomes or lysosomes. Although the exact nature of Pep1-mediated intracellular delivery remains elusive in this study, this process is likely to be micropinocytosis, a lipid raft-dependent and receptor-independent endocytosis, reported as a primary entry route for arginine-rich CPPs [32].

Despite many attempts to develop in vitro assays of MMPs involving the use of fluorogenic peptides, peptide-conjugated proteins [33,34], or peptide-conjugate nanoparticles [15,35,36,37], the imaging of living cells is essential to understand the functional roles of MMPs in cell migration or tissue remodeling. We recently developed a Förster resonance energy transfer (FRET)-based reporter to monitor MMP2 activity in living cells [10], which allowed for the long-term monitoring of MMP2 activity using ECM-sticky probes. While such an ECM-immobilized probe can be useful in defined ECM regions, the development of cell-specific fluorescence probes is required to monitor cell-specific MMP secretion during cell migration. In this regard, protease-activatable CPPs are useful in living cells [28]. Based on our results, one can assume two major pieces of information: (i) the degree of MMP2 activity in cell population over time (this can be estimated by calculating the proportion of fluorescent cells in total naïve cells) and (ii) the relationship between the time-lapse cellular uptake rate and the MMP2 activity (Pep1 showed the cellular uptake of 40~60% in HT-1080 cells for 30 min). However, our method provides distinct advantages over the processes of previous studies. First, compared to peptide probes conjugated with nanomaterials or various FRET couplers, the peptide used in this study had greater versatility in cell imaging because it is simple and convenient to synthesize and use. Second, short fluorescent peptides enable the protease-modulated real-time monitoring of target cells for a brief period of time. In contrast to the current R_4_-included peptides, R_4_-fused fluorescent proteins did not show significant intracellular delivery to HT-1080 cells in our preliminary study due to their relatively larger size (data not shown). This observation supports a previous finding that cationic CPPs with small cargoes are likely to enter cells faster than cationic CPPs with large cargoes [38]. Although real-time monitoring of MMP2 was accomplished in this study, further studies are required to apply this method to other cell types. For example, unlike HT-1080 cells, HeLa cells did not take up cleaved Pep1 or Pep3, meaning that the cationic strength or conformational structure of arginine-rich peptide sequences is presumably dependent on cell type. Cell-specific CPPs have been identified by screening phage display libraries, and they exhibit different sequences and properties by cell type [39]. Classical fluorophores such as FITC could not be used for long-term monitoring (more than a couple of hours) due to their fast photobleaching or pH-dependent decay; however, when coupled with advanced fluorescence imaging [40] or cell-sorting techniques [41], this method can facilitate the rapid monitoring of protease activity in living cells at the single-cell level.

## 3. Materials and Methods

### 3.1. Materials

Fetal bovine serum (FBS), Roswell Park Memorial Institute (RPMI)-1640 culture media, penicillin-streptomycin (P/S), NucBlue, and Lysotracker Red DND-99 were purchased from Thermo Fisher Scientific (Waltham, MA, USA). Goat anti-mouse IgG (H + L) horseradish peroxidase (HRP) conjugate, active MMP2 (No. PF023), and active MMP9 (No. PF024) were purchased from Merck Millipore (Burlington, MA, USA). Human anti-MMP2 antibody (No. MAB9021) was purchased from R&D systems (Minneapolis, MN, USA). Goat anti-rabbit IgG (H + L) HRP conjugate (No. ab6721) and anti MMP9 antibody (No. ab38898) were purchased from Abcam (Cambridge, UK), while 3-(4,5dimethylthiazol-2-yl)-2,5-diphenyltetrazolium bromide (MTT) was purchased from Cell Biolabs (San Diego, CA, USA). Polyvinylidene difluoride membrane and enhanced chemiluminescence detection kits were purchased from GE Healthcare (Chicago, IL, USA). MMP2/9 inhibitor ((2R)-2-[(4-biphenylylsulfonyl)amino]-3-phenylpropionic acid), gelatin from porcine skin, and Triton X-100 were purchased from Sigma Aldrich (St. Louis, MO, USA). SYBR Premix Ex Taq II and PrimeScript™ real-time PCR kits were purchased from Takara Bio (Shiga, Japan). The primers for the RT-qPCR were synthesized by Macrogen (Seoul, Korea). FITC-labeled peptides (Pep1, EEEEGGIPVSLRSGGGRRRRK-FITC; Pep2, EEEEGGRRRRK-FITC; Pep3, GGIPVSLRSGGGRRRRK-FITC) were synthesized by Peptron (Daejeon, Korea).

### 3.2. Cell Culture

Human fibrosarcoma (HT-1080) and colorectal adenocarcinoma cells (HT-29) were obtained from the Korean Cell Line Bank (Seoul, Korea). Typically, the cells were cultured in T-25 tissue culture flasks (SPL, Korea) in RPMI-1640 media containing 10% FBS and 1 × P/S and incubated at 37 °C in a humidified incubator with a 5% CO_2_ atmosphere. When the cells reached 80% confluence, the culture media were changed to fresh serum-free conditioned media. For immunoblotting and zymography, the media were collected 48 h after culture and concentrated by using an Amicon ultra centrifugal filter unit (50 kDa MWCO, Millipore, Billerica, MA, USA) and by centrifugation (1500× *g* for 20 min).

### 3.3. Reverse Transcription-Polymerase Chain Reaction (RT-PCR) and Real-Time PCR

RT-PCR or real-time PCR primers were designed using the MMP2 and MMP9 sequences. The forward primer for MMP2 was 5′-TTT CCA TTC CGC TTC CAG GGC AC-3′, and the reverse primer was 5′-TCG CAC ACC ACA TCT TTC CGT CAC T-3′, which yielded a product with 253 base pairs (bps). The forward primer for MMP9 was 5′-CAC TGT CCA CCC CTC AGA GC-3′, and the reverse primer was 5′-GCC ACT TGT CGG CGA TAA GG-3′, which yielded a 263 bp product. The total mRNA was isolated from two types of cells using Qiagen RNeasy kit (Qiagen, Valencia, CA, USA), and complementary DNA (cDNA) was synthesized from 1 μg of total extracted mRNA using reverse transcriptase (Takara Bio). The RT-PCR was run for 30 s at 95 °C, followed by 40 cycles for 5 s at 95 °C (denaturation) and 30 s at 60 °C (annealing/extension). The PCR products were run on 1.2% agarose in 1× Tris-Borate-EDTA buffer at 100 V for 30 min. The expression levels of the MMP2 and MMP9 genes were compared to those of glyceraldehyde 3-phosphate dehydrogenase (GAPDH, a housekeeping gene with functions in glycolysis). The following forward and reverse primers for GAPDH were used: 5′-GAA GGT GAA GGT CGG AGT-3′ and 5′-GAA GAT GGT GAT GGG ATT TC-3′. Real-time PCR was performed using a CFX connect real-time thermal cycler (BioRad, Hercules, CA, USA) in a total volume of 25 μL with SYBR Green (RR820; Takara Bio) and 2 μL of cDNA as a template. The samples were heated to 95 °C for 3 min, followed by 40 cycles of denaturation at 95 °C for 3 s and annealing at 60 °C for 40 s without an extension step. The cycle quantification value (Cq; the PCR cycle number when the sample reaction curve intersects the threshold line) was determined, and the comparative initial gene expression level was calculated in each sample after normalization on the basis of the expression of GAPDH. 

### 3.4. Western Blot and Zymography

For the Western blot, 20 μg of total protein in a concentrated culture medium (CM) of cells was resolved by 12% sodium dodecyl sulfate-polyacrylamide gel electrophoresis (SDS-PAGE) and transferred to polyvinylidene fluoride membranes (GE Healthcare). After blocking in TBST buffer (50 mM Tris containing 150 mM NaCl and 0.1% Tween-20, pH 7.4) supplemented with 5% non-fat dry milk, the membranes were incubated overnight at 4 °C with anti-MMP2 antibody or anti-MMP9 antibody. These are monoclonal antibodies that can associate with the pro and active forms of MMPs. The membrane was rinsed three times with fresh TBST buffer and incubated for 1 h at room temperature (RT) with 10,000-fold diluted, HRP-conjugated, goat anti-mouse IgG and further treated with Western blotting substrate (GE Healthcare). Signal intensities were quantified using a Fusion SL chemiluminescence image acquisition system (Vilber Lourmat, Torcy, France). For gelatin zymography analysis, the total protein (10 μg) content of the culture medium was separated in the absence of heat or reducing reagent and loaded to 12% SDS-PAGE, including 0.1% (w/v) gelatin (Sigma-Aldrich). The gels were incubated at RT for 1 h in renaturing buffer (50 mM Tris-HCl containing 2% Triton X-100, pH 7.4) and subsequently at 37 °C for 48 h in developing buffer (50 mM Tris-HCl containing 150 mM NaCl and 10 mM CaCl_2_, pH 7.4). The gels were stained with 0.3% Coomassie Brilliant Blue, and proteolysis was detected as a white band against a blue background.

### 3.5. Fluorescent Gel Electrophoresis

Fluorescent gel electrophoresis was performed on 0.8% agarose gel at 50 V for 60 min. The agarose gel was cast and run using 0.5 × TB buffer. For the protease assay, Pep1 or Pep2 (1 μL at 2 mM, final 50 μM) was mixed with active MMP2 or active MMP9 (1 μL, final 0.1 μg) at a final volume of 40 μL in standard reaction buffer (20 mM Tris-HCl containing 5 mM CaCl_2_ and 100 mM NaCl, pH 7.6). The reactants were incubated in a tube for 120 min at 37 °C to induce the enzyme reaction, and 30 μL of the solution were loaded onto the agarose gel. As a control, a solution of Pep1 or Pep 2 (1 μL at 2 mM) without enzymes was resuspended in a reaction buffer (for a total of 40 μL), and the aliquot (30 μL) was used in gel electrophoresis. The fluorescent image of the agarose was captured using a home-built transilluminator equipped with a blue light-emitting diode lamp. The original image underwent a pseudo-color imaging process using an image-editing function in Microsoft PowerPoint 365.

### 3.6. Cell Imaging

For live cell imaging, HT-1080 or HT-29 cells were cultured in Lab-Tek II chambered coverglass (Thermo Fisher Scientific) containers in RPMI-1640 media containing 10% FBS and 1× P/S at 37 °C. When the cells were 80% confluent, the culture medium was rinsed and changed to a fresh serum-free medium, followed by the addition of peptide probes at a final concentration of 10 μM. For organelle images, nuclei and lysosomes (or late endosomes) were stained with NucBlue (1 drop) and Lysotracker Red (50 nM) in serum-free media, respectively, and incubated in the dark for 10 min. After washing of the cells and removal of the chamber, cells adhering to the coverglass were subjected to bright-field or fluorescent images using a confocal microscope (Nikon C2si+, Japan). The excitation/emission wavelengths of NucBlue, FITC, and Lysotracker Red were 352/454 nm, 488/525 nm, and 577/590 nm, respectively. Typically, images were collected using a pixel dwell time of 10.3 μs on a 512 × 512 pixel image with 1 mW light power at the objective through a pinhole (60 μm) to reach the PMT. The frame rate was rather slow; the frame rate was about 0.255 FPS (frame per second) for a 512 × 512 frame (i.e., 1 frame per 3.9 s). To gain time-lapse images for FITC in living cells, one video file was constructed with images taken every 20 s for 30 min. Imaging analysis was performed using the NIS-Elements microscope imaging software (Ver 2.0).

### 3.7. Cell Viability Test

For the cell viability test, HT-1080 cells were treated with Pep1 (final 10 μM) or Pep2 (final 10 μM) over time (0–24 h), which was followed by the incubation with fresh medium containing MTT (0.5 mg mL^−1^) for 2 h at 37 °C. After the culture medium was removed, 100 μL of dimethyl sulfoxide (DMSO; Sigma-Aldrich) was added to each well to induce the reduction of MTT by mitochondrial dehydrogenases in living cells. The optical density in each well was measured at 570 nm using a microplate reader (Varioskan, Thermo Fisher Scientific).

## 4. Conclusions

We demonstrated the real-time monitoring of cell-secreted proteases using fluorescent cell-permeable peptides, including anionic (E_4_), cleavable, and cationic sequences (R_4_). In the absence of MMP2 activity in living cells, the peptides were silenced due to the electrostatic blockage of intracellular delivery. In contrast, the MMP2 activity of living cells promoted the delivery of peptides into the cell via protease cleavage, which led to fluorescence intensity in the cell. As a result, compared to HT-29 cells (MMP2-negative), HT-1080 cells (MMP2-positive) exhibited a strong fluorescence signal response to the short fluorescent peptide (Pep1) via cell-secreted protease activation. Owing to its simplicity and versatility, this approach will be useful for real-time monitoring and the rapid identification of protease activity in living cells.

## Data Availability

Not applicable.

## Supplementary Materials

The following are available online at https://www.mdpi.com/article/10.3390/ijms23031605/s1.
