# Supplementary material for: Activatable Peptides for Rapid and Simple Visualization of Protease Activity Secreted in Living Cells
Source: Int J Mol Sci. 2022 Jan 30;23(3):1605. doi: 10.3390/ijms23031605 (PMC8836073; doi:10.3390/ijms23031605)
Supplement: Supplementary file 1 [file ijms-23-01605-s001.zip › Supplementary Materials.pdf]

Supplementary Material

Activatable Peptides for Rapid and Simple Visualization of Protease Activity Secreted in Living Cells

Gae-Baik Kim[1†], Jeong Min Lee[1†], Duc Long Nguyen[1,2†], Joonseok Lee[3,4,5] and Young-Pil Kim[1,2,4,6]*

[1] Department of Life Science, Hanyang University, Seoul 04763, Republic of Korea; valkyriex@naver.com (G.-B.K.); jeremy1989@naver.com (J.M.L.); quyle.dongphuong@gmail.com (D.L.N.); ypilkim@hanyang.ac.kr (Y.-P.K.)

[2] Research Institute for Convergence of Basic Sciences, Hanyang University, Seoul 04763, Republic of Korea

[3] Department of Chemistry, Hanyang University, Seoul 04763, Republic of Korea; joonseoklee@hanyang.ac.kr (J.L.)

[4] Department of HY-KIST Bio-Convergence, Hanyang University, Seoul 04763, Republic of Korea

[5] Molecular Recognition Research Center, Korea Institute of Science & Technology (KIST), Seoul 02792, Republic of Korea

[6] Research Institute for Natural Sciences, Hanyang University, Seoul 04763, Republic of Korea

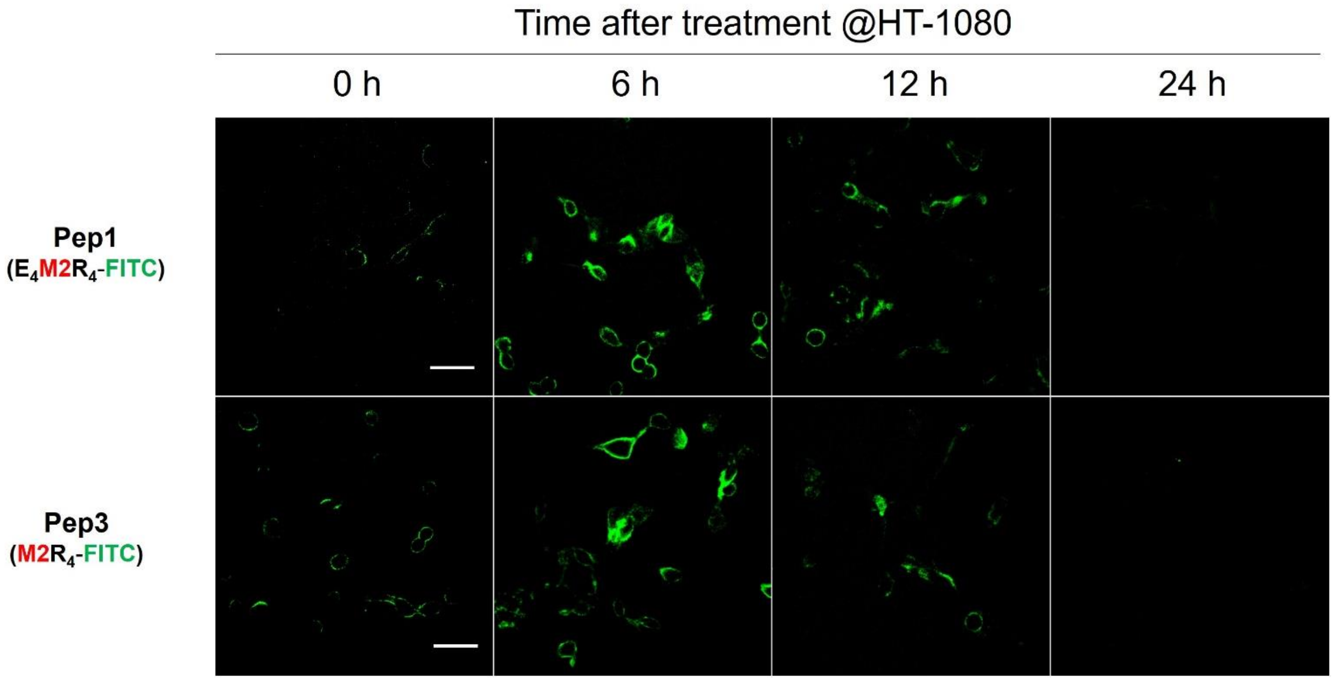

Figure S1. Time-lapse fluorescence image of HT-1080 cells at 37 °C over time (0 to 24 h) after Pep1 (top) or Pep3 (bottom) treatment. Cells were exposed to laser excitation whenever image was taken at a given time. The peptide probe (final 10 μM each) was added to the CM of HT-1080 cells. Scale bar: 50 μm.

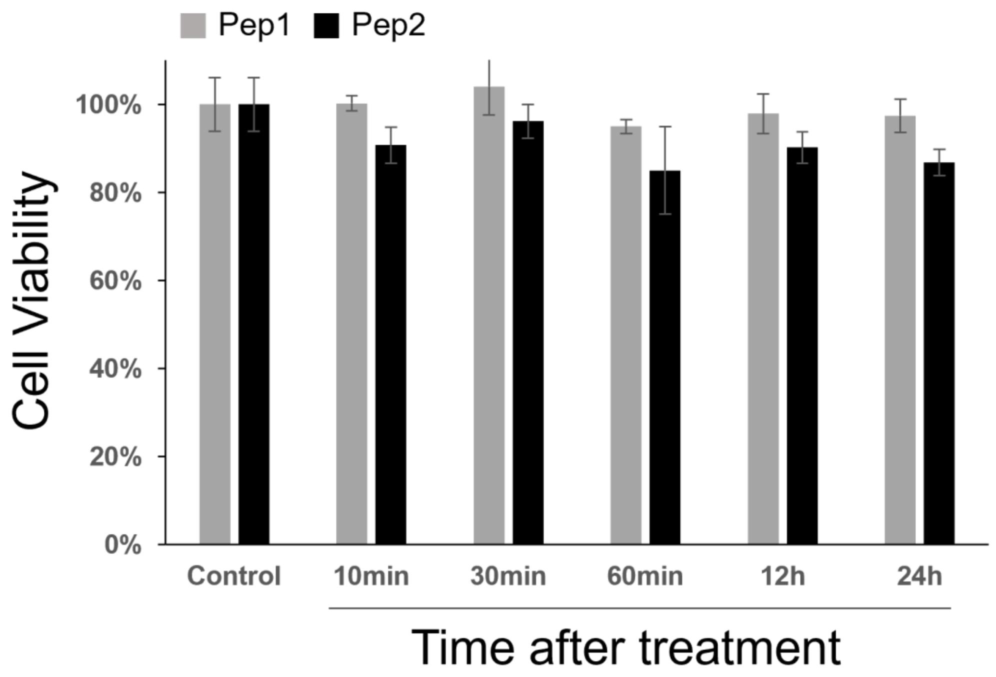

Figure S2. MTT assay to evaluate the cell viability in HT-1080 cells by treatment of Pep1 (gray) or Pep2 (black). Time-dependent viability in the peptide-treated cells was represented as bar graphs. Error bars represent the standard deviations of triplicate experiments.

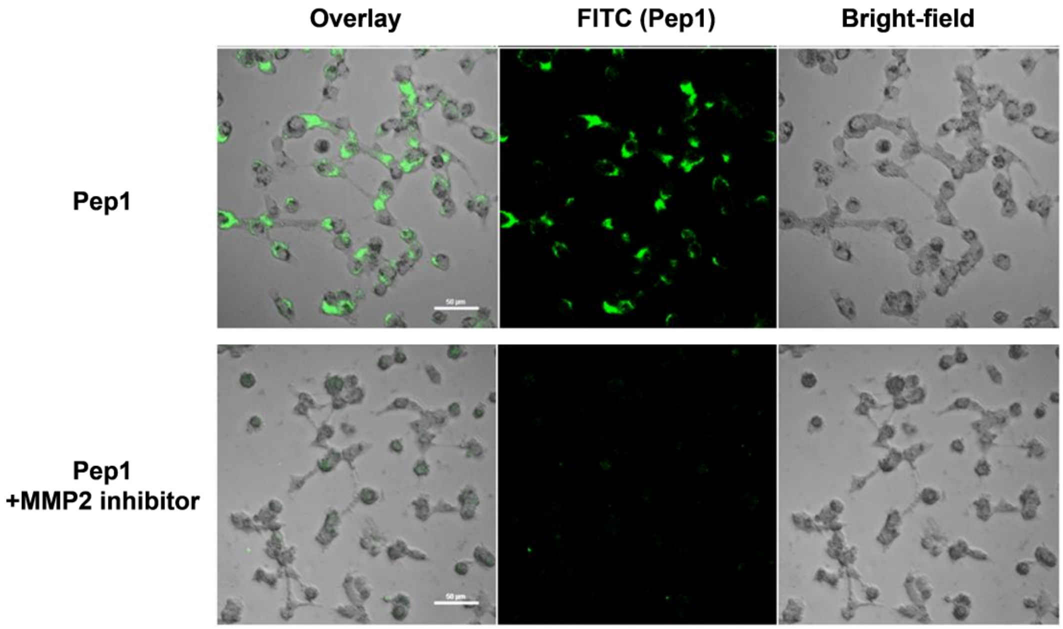

Figure S3. Overlay (left), fluorescence (middle), and bright-field (right) images of MMP2-expressed HT-1080 cells using Pep1 in the absence (top) or presence (bottom) of MMP2 inhibitor. The Pep1 (final 10 μM each) with and without MMP2 inhibitor (final 10 μg mL^{-1}) was added to the CM of HT-1080. Scale bar: 50 μm.

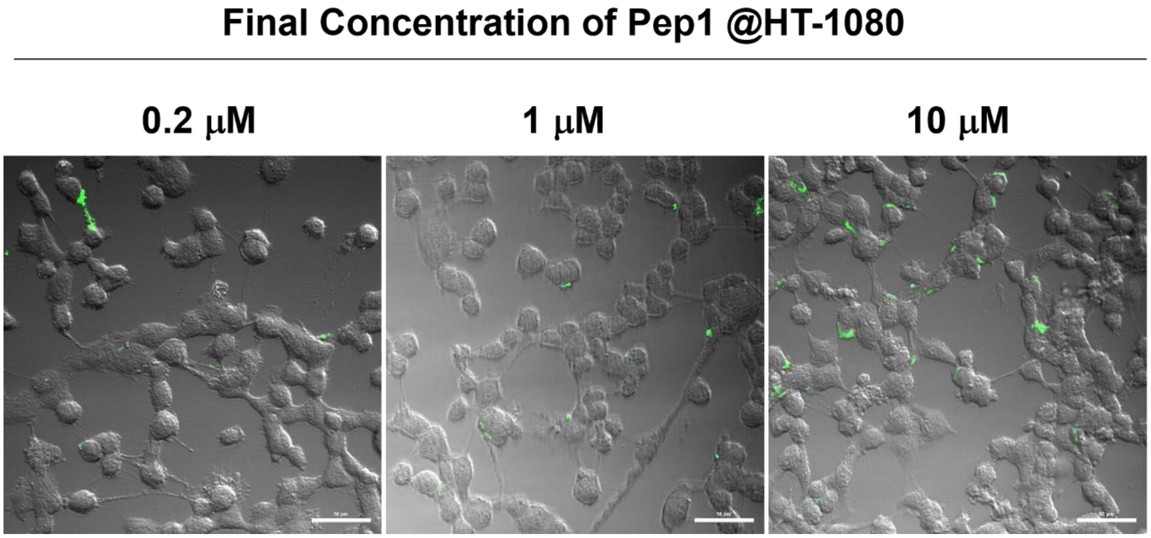

Figure S4. Overlay (bright-field and fluorescence) confocal images of HT-1080 (MMP2-positive cells) treated with different concentrations (0.2, 1, or 10 μM) of Pep1. The Pep1 was added to the serum-free culture media of the cells. Images were collected from 30 min after treatment. Scale bar: 50 μm.

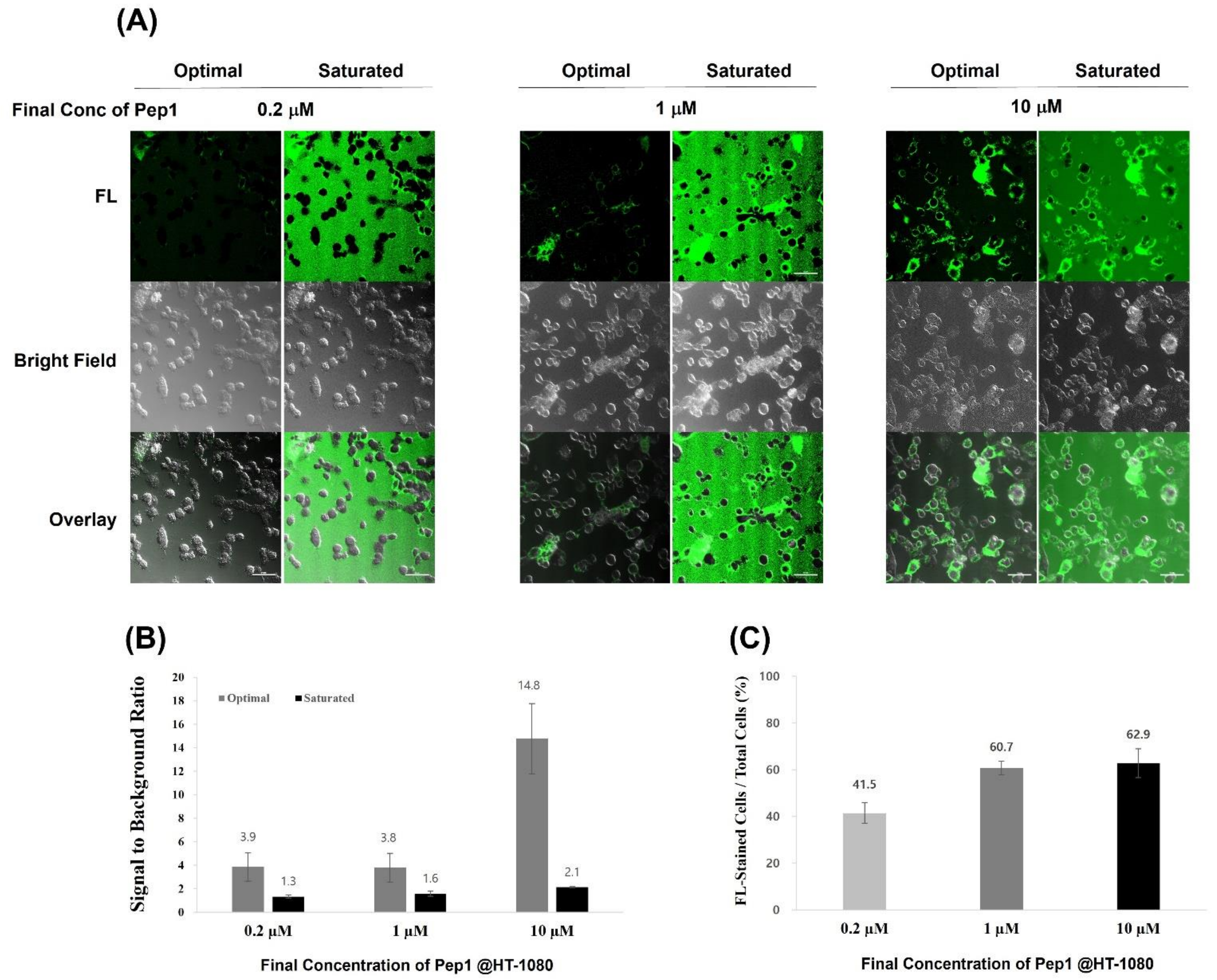

Figure S5. Concentration effect of Pep1 on intracellular imaging and uptake. (A) Fluorescence (FL), bright-field, and overlay images of HT-1080 cells at 37 °C. Images were taken under the optimal or saturated conditions with different laser PMT gain or offset. The Pep1-FITC was treated with cells at each concentration for 30 min. Scale bar: 50 μm. (B–C) Graphs showing imaging analysis from (A): (B) Signal to background ratio as a function of Pep1 concentration. (C) The percentage of FITC-stained cells over total cells in overlay images under optimal laser condition. The error bars indicate three different images (A is the representative image).

Movies S1−S4. Real-time videos of live cells for 30 min after treatment with Pep1.

Movie S1. Real-time fluorescence and bright-field overlay video of live HT-1080 cells for 30 min after treatment with Pep1 (E_4M2R_4-FITC, final 10 μM). The video play is compressed 300 times faster than real time. Scale bar: 50 μm.

Movie S2. Real-time fluorescence video of live HT-1080 cells for 30 min after treatment with Pep1 (final 10 μM). The video play is compressed 300 times faster than real time. Scale bar: 50 μm.

Movie S3. Real-time fluorescence and bright-field overlay video of live HT-29 cells for 30 min after treatment with Pep1 (final 10 μM). The video play is compressed 300 times faster than real time. Scale bar: 50 μm.

Movie S4. Real-time fluorescence video of live HT-29 cells for 30 min after treatment with Pep1 (final 10 μM). The video play is compressed 300 times faster than real time. Scale bar: 50 μm.
